# PANoptosis in cancer, the triangle of cell death

**DOI:** 10.1002/cam4.6803

**Published:** 2023-12-08

**Authors:** Hantao Cai, Mingming Lv, Tingting Wang

**Affiliations:** ^1^ The State Key Laboratory of Pharmaceutical Biotechnology, Division of Immunology, Medical School Nanjing University Nanjing China; ^2^ Jiangsu Key Laboratory of Molecular Medicine, Division of Immunology, Medical School Nanjing University Nanjing China; ^3^ Department of Breast Women's Hospital of Nanjing Medical University, Nanjing Maternity and Child Health Care Hospital Nanjing China

**Keywords:** apoptosis, cancer, necroptosis, PANoptosis, PANoptosome, pyroptosis

## Abstract

**Background:**

PANoptosis is a novel form of programmed cell death (PCD) found in 2019 that is regulated by the PANoptosome. PANoptosis combines essential features of pyroptosis, apoptosis, and necroptosis, forming a “death triangle” of cells. While apoptosis, pyroptosis, and necroptosis have been extensively studied for their roles in human inflammatory diseases and many other clinical conditions, historically they were considered as independent processes. However, emerging evidence indicates that these PCDs exhibit cross talk and interactions, resulting in the development of the concept of PANoptosis.

**Methods:**

In this review, we offer a concise summary of the fundamental mechanisms of apoptosis, pyroptosis, and necroptosis. We subsequently introduce the notion of PANoptosis and detail the assembly mechanism of the PANoptosome complex which is responsible for inducing cell death. We also describe some regulatory networks of PANoptosis.

**Results:**

PANoptosis now has been associated with various human diseases including cancer. Although the exact function of PANoptosis in each tumor is not fully understood, it represents a prospective avenue for cancer therapy, offering promise for advancements in cancer therapy.

**Conclusions:**

In the future, in‐depth study of PANoptosis will continue to help us in understanding the fundamental processes underlying cell death and provide scientific support for cancer research.

## Graphical abstract

Our review describes the basic mechanisms of PANoptosis with its important role in cancer. We provide new directions for the study of oncology drugs.

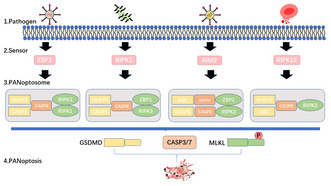

## 1 INTRODUCTION

Cell death is a fundamental process in the normal growth and development of human body, as well as for its immune defense against pathogenic invasions.^1^ Initially, researchers classified cell death into accidental cell death (ACD) and programmed cell death (PCD) based on morphological changes in cells and the status of DNA fragmentation. According to the redefinition by Nomenclature Committee on Cell Death in 2018, PCD is now considered a strictly regulated form of cell death occurring under physiological conditions, referred to as regulatory cell death (RCD).^2^ Programmed cell death facilitates the removal of damaged or infected cells, thereby preventing the propagation of harmful agents and preserving tissue homeostasis.^1^, ^3^, ^4^

Of all the programmed cell deaths that have been identified so far, apoptosis, pyroptosis, and necroptosis are the three with the more well‐developed mechanisms and broader physiological functions. Each of the three PCDs possesses distinct molecules that are responsible for triggering and carrying out cell death through varying mechanisms.^5^, ^6^, ^7^, ^8^, ^9^, ^10^ In brief, apoptosis is a complex cell death process activated under the regulation of cellular genes, involving a cascade response of the caspase family. It can be initiated by both intrinsic and extrinsic pathways that activate downstream caspase‐9 and caspase‐8, respectively. Then caspase‐9 and caspase‐8 act as cleavage proteins and activate the final death‐executing proteins caspase‐3 and caspase‐7.^11^, ^12^ Pyroptosis serves as a host defense mechanism when confronted with pathogenic infections. It is characterized by a sustained increase in cell volume until the cell membrane ruptures, resulting in the release of cellular contents and the initiation of a robust inflammatory response. Pyroptosis activates the GSDM family through caspase‐1‐dependent formation of inflammasome or directly through caspase‐11 (in mice) and caspase‐4/5 (in human). The ability of GSDM family proteins to create pores in the cell membrane is known to induce cell death and trigger the release of pro‐inflammatory cytokines, including IL‐1β and IL‐18.^13^, ^14^ Necroptosis is an alternative programmed cell death mode that occurs when the normal apoptosis pathway is blocked, displaying features that encompass elements of both apoptosis and necrosis. It activates the substrate MLKL via RIPK1‐RIPK3, which, when phosphorylated, can disrupt the cell membrane and execute death.^15^

For a long time, the three PCDs were thought to be independent of each other, performing distinct physiological functions in different contexts. However, there is growing evidence of cross talk and interactions between apoptosis, pyroptosis, and necroptosis.^16^, ^17^ Therefore, American scholar Malireddi et al. proposed a novel notion of programmed death: PANoptosis in 2019.^18^ PANoptosis possesses the key molecular and functional characteristics that are shared by pyroptosis, apoptosis, and necroptosis, yet it cannot be fully explained or generalized by any of these individual pathways alone. The regulation of PANoptosis involves a cascade of upstream receptors and molecular signals that assemble into a complex, PANoptosome. This complex serves as a launching pad for the activation of downstream molecules and a “master switch” for the initiation of all three PCD pathways.^19^ In summary, PANoptosis is like a triangle of cell death, with the three dots of the triangle representing pyroptosis, apoptosis, and necroptosis, and the lines between the vertices representing the interactions between the three PCDs. The role of PANoptosis in infectious, auto‐inflammatory, and metabolic diseases has been widely studied and recognized.^20^ As our knowledge of the contribution of cell death to cancer continues to evolve, PANoptosis is attracting increasing attention as a potential target for intervention in tumor therapy in near future.

This review begins an introduction to the basic mechanisms of apoptosis, pyroptosis, and necroptosis. Additionally, we highlight the cross talk and interactions between these three PCDs. We then discuss the concept and mechanisms of PANoptosis in detail and describe the specific assembly mechanisms of the four currently known PANoptosome, which are responsible for inducing cell death. Next, we describe the currently known regulatory mechanisms of PANoptosis. Finally, we will highlight the importance of PANoptosis in cancer and look at the implications and feasibility of treating cancer by targeting molecules or proteins that interfere with PANoptosis, which we hope may provide some inspiration to researchers in related fields.

## 2 APOPTOSIS, PYROPTOSIS, NECROPTOSIS, AND CROSS TALK

### 2.1 Apoptosis

#### 2.1.1 Morphology of apoptosis

Apoptosis was introduced and described in 1972 as the first form of programmed cell.^21^ In general, apoptosis refers to the programmed cell death of cells under certain physiological or pathological conditions, mediated by caspase protein hydrolases. Apoptosis exhibits characteristic changes in morphology when observed under the electron microscope. The cell nucleus becomes condensed and smaller, with chromatin condensing into nucleosomes due to DNA degradation. The cytoplasm undergoes significant changes, becoming more solid and forming numerous apoptotic bodies. Additionally, the cell membrane surface develops membrane blebs, which can be engulfed and degraded by neighboring cells or macrophages. During apoptosis, the structure inside the mitochondria becomes looser, and the morphology of the mitotic spindle changes.^2^, ^22^

#### 2.1.2 Mechanism of apoptosis

**FIGURE 1:**
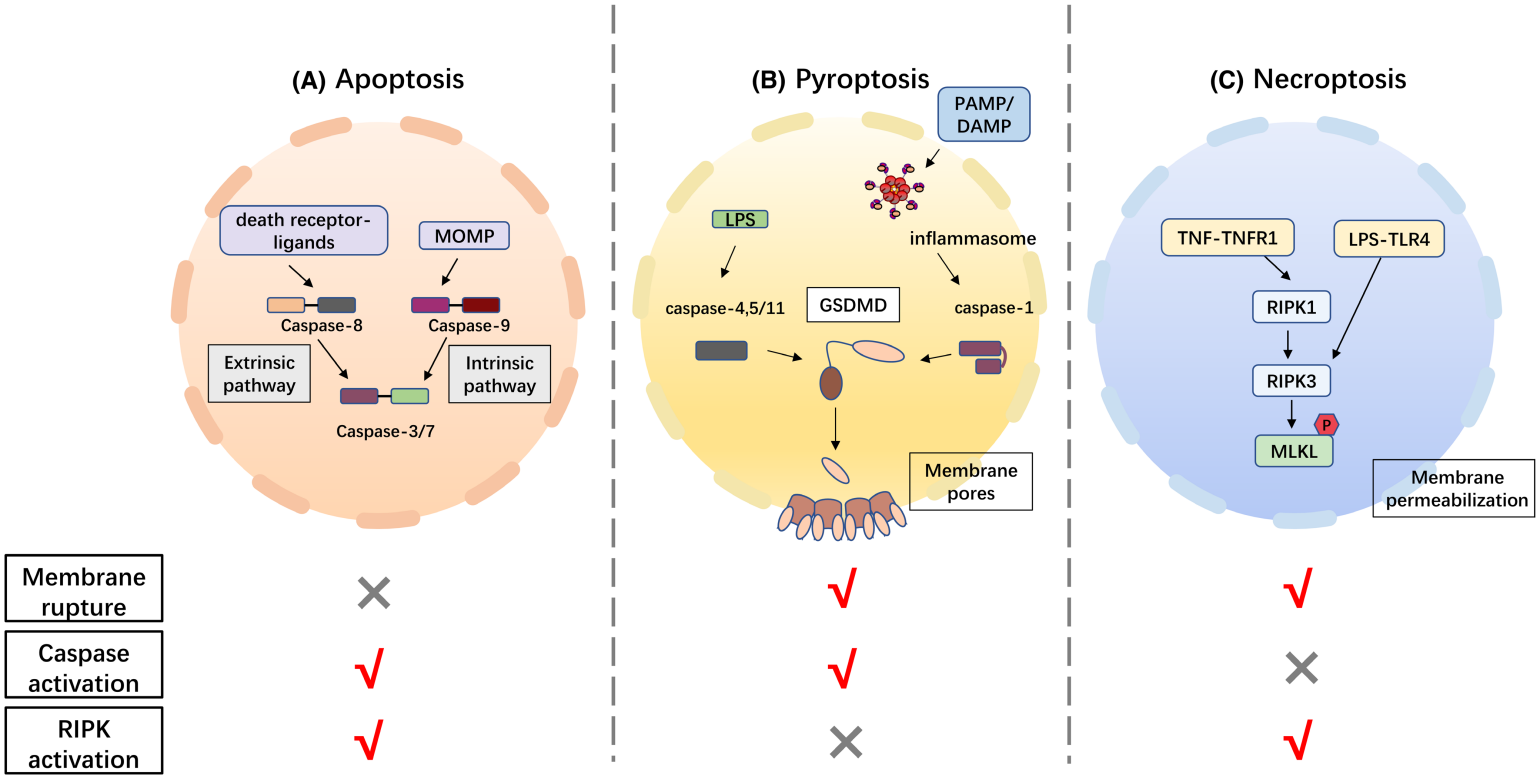
Overview of the three PCD mechanisms. (A) Apoptosis is divided into intrinsic and extrinsic pathways that activate the execution protein caspase‐3/7 via the cleavage protein caspase‐9 and caspase‐8, respectively. (B) Pyroptosis activates the GSDM family through caspase‐1‐dependent formation of inflammatosome or directly through caspase‐11 (in mice) and caspase‐4/5 (in human), forming pores in the cell membrane and induce cell death. (C) Necroptosis activates the substrate MLKL via RIPK1‐RIPK3, which, when phosphorylated, can disrupt the cell membrane and execute death.

In general, apoptosis is divided into intrinsic and extrinsic pathways. The two pathways are initiated differently upstream but share the same final death execution process (Figure 1A).

The intrinsic apoptosis pathway, also called the mitochondria‐dependent pathway, is triggered by intracellular stressors such as DNA damage or oxidative stress, which cause mitochondrial outer membrane permeabilization (MOMP).^23^, ^24^ MOMP is regulated by BCL‐2 family proteins and triggers the release of cytochrome C from the intermembrane space of mitochondria. Upon entering the cytoplasm, cytosolic cytochrome C binds to apoptotic protease‐activating factor 1 (APAF‐1), which interacts with pro‐caspase‐9 through its recruitment domain (CARD) to form a complex called apotosome. This complex activates pro‐caspase‐9 to its mature form caspase‐9, which can then cleave downstream pro‐caspase‐3 and pro‐caspase‐7, allowing them to perform their executioner functions.^25^, ^26^, ^27^, ^28^, ^29^

The extrinsic apoptosis pathway is the receptor‐mediated pathway, which was found shortly after the intrinsic pathway was identified. Members of the transmembrane death receptor family, including the tumor necrosis factor receptor (TNFR), fas ligand receptor (FASL), and TNF‐related apoptosis‐inducing ligand (TRAIL) receptor DR4/5, bind to their corresponding ligands to start the extrinsic pathway.^30^, ^31^ The recruitment of, FAS‐associated protein containing death domain (FADD), RIPK1 and caspase‐8 occurs when the death receptor binds to the corresponding ligand. Central to this process is the homotypic interactions between the two death effector domains of caspase‐8 and FADD. After being recruited, the pro‐caspase‐8 proceeds through auto‐processing to become mature caspase‐8, which cleaves the pro‐forms of caspase‐3/7.^32^, ^33^

Briefly, the intrinsic pathway relies on caspase‐8, which activates the final execution effector caspase‐3/7, whereas the extrinsic pathway relies on caspase‐9. Caspase‐3/7 have similar substrate and inhibitor specificities, and their heterologous activation degrades PARP, DFF‐45 (DNA fragmentation factor‐45), which inhibits DNA repair and the initiation of DNA degradation.^33^, ^34^ Interestingly, the two pathways are not completely isolated. There are several signaling pathways connecting intrinsic and extrinsic apoptosis. Certain type II cells undergo death through an extrinsic pathway requiring Bid cleavage by caspase‐8 to trigger intrinsic mitochondria‐dependent signaling for eventual apoptosis.^35^ Moreover, the extrinsic death receptor signaling can be boosted by the secondary mitochondrial activator of caspase inhibiting cIAP.^36^ These support a model in which the apoptosis signal triggers the simultaneous activation of both branches of apoptosis network once threshold levels are reached, despite caspase initiation being isolated by different pathways.

### 2.2 Pyroptosis

#### 2.2.1 Morphology of pyroptosis

Pyroptosis is the programmed cell death mediated by the gasdermin family depends (not always) on inflammatory caspases, mainly caspase‐1/4/5/11. It is coupled with the release of large amounts of pro‐inflammatory factors like IL‐1β and IL‐18.^13^ Triggers of pyroptosis include bacteria, viruses, and their products, that is, the LPS and DNA of the viruses.^37^ Cells undergoing pyroptosis exhibit characteristic morphological features when observed under electron microscopy, including cell swelling and deformation, rupture and lysis of the cell membrane, mitochondrial expansion and breakdown, disruption and dissolution of the endoplasmic reticulum and Golgi apparatus, appearance of vacuoles, and leakage of intracellular contents such as enzymes, proteins, and nucleic acids.^38^

#### 2.2.2 Mechanism of pyroptosis

The signaling pathways that activate cell pyroptosis include canonical pathway (caspase‐1 dependent), noncanonical pathway (caspase‐4, caspase‐5, and caspase‐11 dependent), and other newly identified pathways.^39^ GSDM family proteins, mainly GSDMD, function as execution proteins for cell death in pyroptosis. When GSDMD is activated by upstream pathways, its N‐terminal will dissociate from the C‐terminal, undergo oligomerization and form pores in the cell membrane that allow large amounts of water to get inside the cells, inducing cell death as well as inflammatory responses^40^, ^41^, ^42^, ^43^, ^44^ (Figure 1B).

The canonical pathway depends on the complex inflammasome, which detects extracellular stimulus signals, recruits and activates caspase‐1. Then the N‐terminal sequence of the GSDMD is cut by activated caspase‐1, allowing it to carry out the executive function of cell death. In addition, activated caspase‐1 also causes the activation of inflammatory factors such as IL‐18 and IL‐1β, triggering an inflammatory response.^45^ Central to this pathway is the formation and functioning of inflammasome.

The multiprotein complex Inflammasome involves pattern recognition receptors (PRRs), which include the NOD‐like receptor (NLR) or AIM‐like receptor (ALR) family of receptor proteins. PRRs recognize pathogen‐associated molecular patterns (PAMPs) or host‐derived danger‐associated molecular patterns (DAMPs) that are specific to corresponding inflammasome, which in turn trigger downstream signals. Five major inflammasome have been identified, NLRP1, NLRP3, NLRC4, AIM2, and Pyrin, both named after their receptor proteins.^46^, ^47^, ^48^, ^49^, ^50^, ^51^ As mentioned earlier, pyroptosis is a host defense mechanism in response to pathogenic infections. Therefore, the correct recognition of external pathogens by inflammasomes is crucial. For example, NLRP1 can detect lethal toxins from *Bacillus anthracis* and components of Toxoplasma gondii.^46^, ^47^ Of these, NLRP3 is the most intensively studied. Unlike other inflammasomes that are activated by a few highly specific stimuli or only one, NLRP3 inflammasome can react to a wide variety of stimuli that are structurally and chemically unrelated, such as pathogens, toxins, metabolites, and nucleic acids.^52^ NLRC4 can recognize flagellin and type III secretory system proteins. AIM2 recognizes and binds autologous or heterologous negative double‐stranded DNA through the C‐terminal HIM200 domain.^50^ Different from other inflammasome, Pyrin does not directly recognize and bind pathogenic microorganisms; the Pyrin receptor protein senses phosphorylation modification and inactivation of the Rho protein family in host cells by bacterial toxins and thus activates the assembly of inflammasome which attracts ASC as well as pro‐caspase‐1 downstream.^51^

In noncanonical pathway, human caspase‐4, caspase‐5, and mice caspase‐11 are triggered by bacterial lipopolysaccharide (LPS) via the N‐terminal CARD. Functioning as caspase‐1 in canonical pathway, activated caspase‐4/5/11 cleaves and activates GSDMD, inducing pyroptosis. Moreover, in the presence of NLRP3 and ASC, caspase‐4 activates caspase‐1 to cleave the pro‐forms of IL‐1β and IL‐18, forming their mature forms.^14^, ^53^

### 2.3 Necroptosis

#### 2.3.1 Morphology of necroptosis

Necroptosis, also called programmed cell necrosis, is a caspase‐independent mode of programmed cell death. Necroptosis is usually characterized under electron microscopy by cell swelling and enlargement, cell membrane rupture and dissolution, disruption and dissolution of internal organelles and tissue structures, leakage of cytoplasmic contents, fragmentation and disintegration of the cell nucleus, as well as an inflammatory response.^10^ Necroptosis, as a mode of cell death intermediate between apoptosis and necrosis, has some key morphological features that can be used to distinguish it from the two. Cells undergoing necroptosis experience swelling, which can be used to differ from apoptosis. And one key morphological feature of necroptosis is unclear consideration (pyknosis) that can help us to identify it from necrosis.

#### 2.3.2 Mechanism of necroptosis

Necroptosis will be initiated by members of the TNF receptor superfamily, including TNFR1, Fas, TRAILR1/2 and DR6, TLR3/4, interferon receptors, and ZBP1.^54^ The main molecules involved in necroptosis‐related signaling include RIPK1, RIPK3, and its substrate, mixed lineage kinase like (MLKL).^55^ Interestingly, although necroptosis was initially found to be inextricably linked to RIPK1 activation, it has now become clear that RIPK3 phosphorylates MLKL and induces its migration to the cell membrane by forming multimers, serving as the key molecular mechanism of necroptosis.^56^ Activation of RIPK3 can occur through either RIPK1‐dependent or RIPK1‐independent pathways (Figure 1C).

The TNF‐initiated, RIPK1‐ and RIPK3‐mediated pathway is the most classical one in necroptosis. When apoptotic caspase‐8 is absent or inhibited, the stimulation of receptors can induce necroptosis. RIPK1 recruits downstream RIPK3 and MLKL to form necrosome. Then the executor protein MLKL will be phosphorylated by RIPK3 and form oligomers. MLKL oligomers can insert into the cell membrane, binding to lipid phosphatidylinositol and cardiolipin. This binding disrupts the integrity of the cell membrane, creating pores that enable the release of intracellular contents.^15^, ^57^, ^58^ In addition, the MLKL‐mediated inward flow of calcium and sodium ions is also involved.^59^

TLR3/4 of the Toll‐like receptor family are also known to induce necroptosis when caspase‐8 activity is inhibited. More specially, TLR3 and TLR4 possess a signaling pathway connected by TIR domain‐containing adapter‐inducible interferon B (TRIF), which allows them to assemble directly with RIPK3 and phosphorylate downstream MLKL, inducing necroptosis independent of RIPK1.^60^, ^61^

### 2.4 Cross talk between apoptosis, pyroptosis, and necroptosis

The three modes of programmed death, apoptosis, pyroptosis, and necroptosis form the triangle of cell death. Although these three pathways have their own distinct activation pathways, execution proteins and modalities of regulation, growing evidence suggests that they are not completely independent. The first molecule identified to associate apoptosis and pyroptosis was caspase‐1. It originally functions in pyroptosis, responsible for activating the downstream death executor protein GSDMD. However, in the absence of GSDMD, caspase‐1 can cleave caspase‐3/7 in apoptosis.^62^, ^63^ Caspase‐3 and GSDME are regulators between apoptosis and pyroptosis. Caspase‐3 can cleave GSDME and induce pyroptosis, while GSDME can also feed forward upstream to control caspase‐3 activation and induce apoptosis.^64^, ^65^, ^66^ Caspase‐8 is another key protein between apoptosis and pyroptosis. It has been found to take part in the onset of pyroptosis in many ways like activating GSDMD directly or participating in the assembly of inflammasome.^67^, ^68^, ^69^

The relationship between pyroptosis and necroptosis mainly focuses on the mutual regulation of the typical NLRP3 inflammasome with RIPK3/MLKL in necroptosis. Recent studies show that NLRP3 inflammasome as an initiator of pyroptosis can also activate the final execution protein of necroptosis, MLKL.^70^, ^71^ Similarly, NLRP3 inflammasome is activated by RIPK3 and MLKL under certain conditions.^70^, ^72^

Necroptosis was once considered an alternate pathway to caspase‐8‐dependent apoptosis, mediating cell death when caspase‐8 was inhibited. Although it has been shown that necroptosis has other distinct physiological implications, the role of caspase‐8 in switching between necroptosis and apoptosis remains decisive.^70^, ^73^ RIPK1, a molecule common to both necroptosis and apoptosis cell death pathways, functions as a regulatory role between the two.^74^ In addition, RIPK3 also determines the direction of cell death: apoptosis or necroptosis.^75^

## 3 PANOPTOSIS

### 3.1 Overview and definition

**FIGURE 2:**
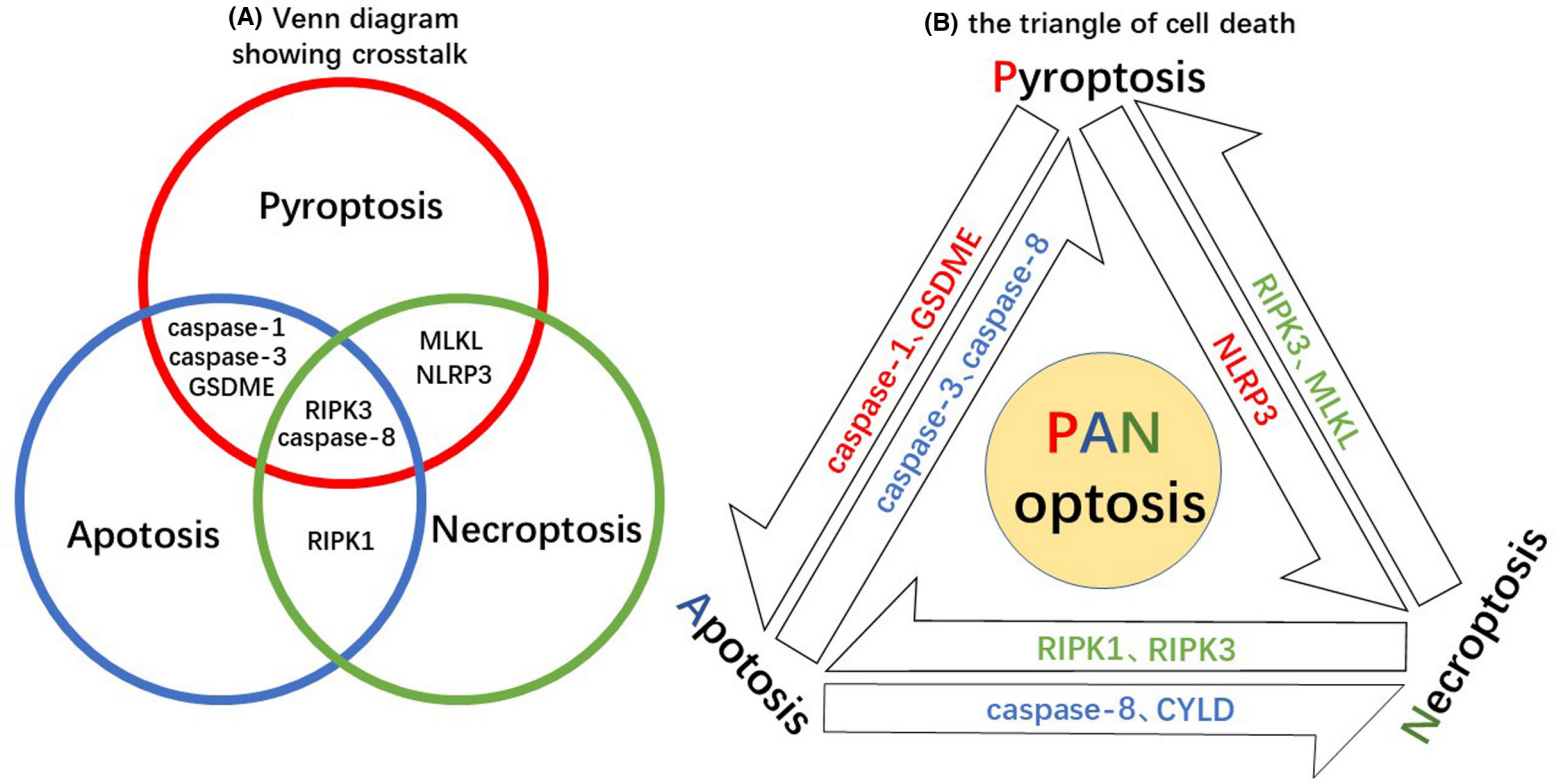
The triangle of cell death. (A) Venn diagram illustrates the cross talk among the three cell death pathways. The overlapping regions between two circles represent the cross talk between two PCD pathways, while the intersection of the three circles in the center represents molecules that play a role in all three PCD pathways. (B) Pyroptosis, apoptosis, and necroptosis form the three vertices of the cell death triangle. The molecules on the sides of the triangle are some of the key molecules that are regulated by the two of these three. Together, these form PANoptosis, which triggers cell death.

Although apoptosis, pyroptosis, and necroptosis were historically considered to be mutually independent of programmed cell death, cross talk between the three death pathways described above has demonstrated extensive mechanistic overlap and interactions between them, from pathway initiators to death execution proteins^19^ (Figure 2A). It has been found that activation of caspase‐1, caspase‐3/8, and MLKL, proteins essential for pyroptosis, apoptosis, and necroptosis, respectively, occurs together when macrophages infected with influenza A virus (IAV) undergoing death. Blocking one of these death pathways alone does not rescue cells from death, but deletion of *Zbp1*, which has previously been shown to be a receptor for IAV, can successfully allow cells to survive.^76^, ^77^ In addition to infection, in terms of in vivo genetic experiments, intestinal inflammation occurring in mice with inactivated caspase‐8 enzymes could only be repaired by simultaneous knockdown of *Mlkl* and *caspase‐1* together.^78^, ^79^ These studies all demonstrate that the three cell death pathways are not three parallel pathways but rather form a complex network structure that regulates cell fate. Taken together, the linkage between apoptosis, pyroptosis, and necroptosis contributed to the concept of PANoptosis, which was first proposed by researchers in 2019.^18^ PANoptosis is an inflammatory programmed cell death that has the key features of apoptosis, pyroptosis, and necroptosis, but cannot be characterized by any of these modes of death in isolation. It is also the origin of the term “PANoptosis”, where “P” stands for pyroptosis, “A” for apoptosis, and “N” for necroptosis, collectively forming the triangle of cell death^80^, ^81^ (Figure 2B).

**FIGURE 3:**
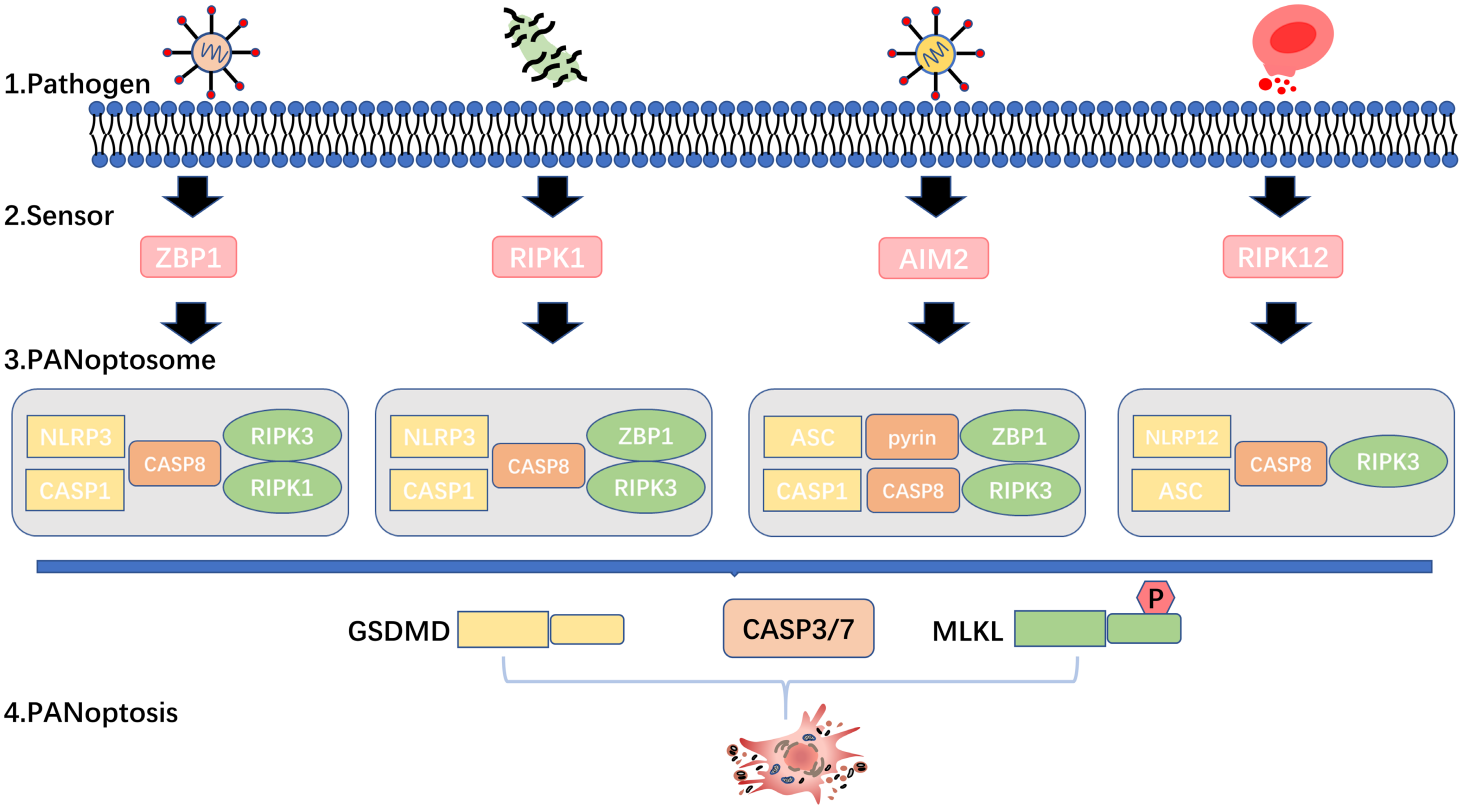
Mechanism of PANoptosome assembly. (1) Different pathogens invade the organism. (2) Specific receptors are activated. (3) Complexes containing key molecules in PANoptosis form PANoptosome, activating three death pathway execution proteins. (4) PANoptosis occurs and cells die.

PANoptosome regulates PANoptosis, just as apoptosome, inflammasome, and necrosome do in their respective death pathways. PANoptosome is initiated by different upstream receptors sensing the corresponding stimuli and acts as a molecular scaffold, allowing the assembly and coupling of important proteins in pyroptosis, apoptosis, and necroptosis through homo‐ or heterotypic interactions, ultimately inducing cell death^82^ (Figure 3). It is worth mentioning that the specific recognition of pathogenic microbial infections by upstream receptors and the exact mechanisms of interaction between these components remain unknown. Four upstream molecules of the PANoptosome have been identified, namely ZBP1, AIM2, RIPK1, and NLRP12, but there are undoubtedly other upstream molecules that initiate the assembly of PANoptosome and induce PANoptosis.^18^, ^83^, ^84^

### 3.2 PANoptosome

#### 3.2.1 ZBP1 PANoptosome

ZBP1 PANoptosome was the first PANoptosome to be identified.^18^ ZBP1, Z‐DNA binding protein 1, contains two Zα domains and a RHIM domain. The two Zα domains (Zα1 and Zα2) play the role of recognizing and binding two left‐handed double helix structured nucleic acid molecules, Z‐DNA and Z‐RNA, respectively.^85^ The RHIM domain in the middle facilitates interactions with other proteins harboring the same domain. ZBP1, by detecting the presence of viral RNA through its Zα structural domain, orchestrates PANoptosis as a defense mechanism against viral infections, including IAV and HSV1. Within this process, Zα domains, especially Zα2 domain, assume a pivotal role in activating proteins associated with programmed cell deaths (PCDs). Notably, the absence of Zα domains or the Zα2 domain alone will fail ZBP1‐mediated cell death following IAV infection.^33^ After stimulation, ZBP1 will form PANoptosome which ultimately leads to lysogenic inflammatory cell death. Current studies have demonstrated that the ZBP1 PANoptosome includes components such as ZBP1, RIPK1, RIPK3, caspase‐8, caspase‐6, caspase‐1, ASC, and NLRP3.^86^, ^87^ ZBP1 recruits RIPK3 through homotypic interaction between RHIM domains, while RIPK3 phosphorylates the downstream necroptosis executive protein MLKL on the one hand and recruits RIPK1 through homotypic interaction between RHIM domains as well on the other. Then RIPK1 binds FADD via the DD end; the DED end of FADD can then recruit caspase‐8, which also contains the DED domain. Caspase‐8 functions as an essential protein of the intrinsic apoptosis pathway and activates the downstream execution proteins, caspase‐3 and caspase‐7. ASC is a central component of the PANoptosome, which associates with caspase‐8 through heterotypic interaction and further recruits NLRP3 and caspase‐1, which are indispensable components for the activation of the pyroptosis execution protein GSDMD.^79^, ^88^, ^89^ In addition, some auxiliary proteins that are not directly involved in pyroptosis, apoptosis, and necroptosis may also have a role in the assembly of PANoptosome. For example, caspase‐6 facilitates the binding between RIPK3 and ZBP1 through interacting with RIPK3 and has a role in ZBP1‐mediated activation of PANoptosome and cell death.^77^ The cell death executors MLKL, caspase‐3/7, and GSDMD are all activated during the formation of the PANoptosome to drive PANoptosis, ultimately leading to lysis‐inflammatory cell death. The specific mechanisms by which these “executioner” proteins perform death have been discussed in detail in the respective death pathways.

#### 3.2.2 RIPK1 PANoptosome

RIPK1 PANoptosome was identified during *Yersinia* infection. RIPK1 is known to be important in necroptosis through its kinase structural domain. However, RIPK1 can also act as a receptor for *Yersinia* with its RHIM domain independent of its kinase activity.^90^ Prof. Malireddi et al. showed that *Yersinia* can initiate RIPK1‐medicated PANoptosis by synergistically activating proteins associated with pyroptosis, apoptosis, and necroptosis. The components of RIPK1 PANoptosome closely resemble those of ZBP1 PANoptosome, with the main distinction lying in the sensor. In this case, RIPK1 serves as the sensor instead of ZBP1. After detecting Yersinia, RIPK1 recruits ASC, NLRP3, ZBP1, caspase‐8, and other proteins through homotypic or heterotypic interactions. The complete assembly procedure closely mirrors that of ZBP1.^84^ Interestingly, unlike ZBP1‐mediated PANoptosis, deleting the sensor RIPK1 did not completely prevent cell death. The deletion of the Ripk1 abolished *Yersinia*‐induced cell pyroptosis and apoptosis but increased the occurrence of ZBP1‐mediated necroptosis. This suggests functional redundancy of some molecules in the RIPK1‐PANoptosome.

#### 3.2.3 AIM2 PANoptosome

AIM2 PANoptosome has been recently reported, whose component molecules include AIM2, ZBP1, pyrin, ASC, caspase‐1, caspase‐8, RIPK3, and FADD.^84^ AIM2 Inflammasome is an important sentinel of innate immune defense, sensing double‐stranded DNA and performing an important part in human development, infectious diseases, inflammation, and tumors. Herpes simplex virus 1 (HSV1), a double‐stranded DNA virus that causes recurrent lifelong incurable lesions, and *Francisella*, a Gram‐negative bacterium that is rapidly lethal after infection, are two different pathogens that activate AIM2, triggering the assembly of AIM2 PANoptosomes and the onset of PANoptosis.^91^, ^92^ In AIM2 PANoptosome, AIM2 acts as an upstream receptor for pyrin and ZBP1, inducing pyrin and ZBP1 transcription in an IFN type I signaling‐dependent manner, thereby increasing their expression levels.^19^ In addition, HSV1 and *Francisella* can inhibit the activity of Rho‐GTP, which was shown to inhibit pyrin activation, and HSV1 and *Francisella* are also involved in the Zα structural domain of ZBP1. This may also account for the fact that the AIM2 PANoptosome contains pyrin and zbp1. ZBP1 is activated and recruits RIPK3 and MLKL downstream in necroptosis, and pyrin activates caspase‐1 in pyroptosis and recruits caspase‐8 in apoptosis via ASC. AIM2, ZBP1, and pyrin each play a role in completing the assembly of PANoptosome and inducing cell death.

#### 3.2.4 NLRP12 PANoptosome

NLRP12 PANoptosome is a newly discovered PANoptosome. NLRP12 is a member of the NLR protein family which has previously only been found to act as an inflammasome sensor during *Yersinia pestis* or *Plasmodium chabaudi* infections, so its role in programmed cell death has been underappreciated.^93^, ^94^ Latest studies this year have uncovered NLRP12 as an innate immune cytoplasmic receptor responsible for heme‐ and PAMPs‐induced activation of inflammasome and PANoptosome that drives cell death under lysogenic conditions. NLRP12 PANoptosome includes NLRP12, caspase‐8, ASC, and RIPK3 four important programmed cell death proteins. Its upstream TLR2 and TLR4‐induced signals upregulate *Nlrp12* expression. However, the specific assembly mechanism of the NLRP12 PANoptosome and the presence of other auxiliary proteins require more in‐depth studies. Moreover, the deletion of *Nlrp12* protected mice from acute kidney injury and death, further suggesting a role for NLRP12 in disease pathogenesis and emphasizing the therapeutic promise of targeting NLRP12 and the molecules involved in its activation pathway for the treatment of various diseases.^95^

#### 3.2.5 Other potential PANoptosomes

Based on the above four types of PANoptosome, it is not difficult to summarize some common features of PANoptosomes. The receptor is the most important part of PANoptosome. Receptors generally require two functional domains: one for specific recognition of pathogens or extrinsic stimuli and the other for interaction with core proteins in PANoptosome. As an example, the Zα domain of ZBP1 is responsible for binding Z‐NA, and the RHIM domain is used to interact with RIPK3. Once a specific protein or proteins have been recruited by the receptor, they can bind and couple to other molecules in PANoptosis through homo‐ or heterotypic interactions, eventually assembling into PANoptosome. Thus, any receptor containing a structural domain that can homotypically interact with molecules in PANoptosis, such as RHIM, PYD, CARD, and DED, may induce the assembly of PANoptosome upon activation by a specific stimulus.

RIG‐1 is one such potential receptor for PANoptosome. During Sendai virus (SeV) infection, RIG‐1 acts as a viral receptor that forms a complex with CASP8 and RIPK1. This may be related to the CARD‐CARD homotypic interaction between RIG‐1 and other molecules containing the CARD domain in PANoptosis.^96^, ^97^ However, there is no definitive conclusion and more in‐depth studies are needed. Another study found that TRIF containing the RHIM domain could mediate the formation of a cell death complex containing RIPK1, FADD, and caspase‐8. This may be closely linked to the ability of TRIF to interact with RIPK3, which also contains the RHIM structural domain, although direct evidence for this is lacking.^98^, ^99^

In conclusion, it is impossible for there to be only the four types of PANoptosomes introduced above. There are still many different PANoptosomes mediated by receptors in different conditions that are unknown and await discovery by researchers.

## 4 REGULATION OF PANOPTOSIS

PANoptosis, as a type of programmed cell death, is also tightly regulated in the body. Uncontrolled cell death will be very harmful to the organism. Therefore, the regulation of PANoptosis is very important for the protection of the human system. In terms of the mechanism of PANoptosis, its regulation can be mainly divided into three parts: recognition of DAMPs and PAMPs, formation of PANoptosome, and activation of execution proteins.^100^ However, due to the diversity of both pattern recognition receptors on the cell surface and death execution proteins, it is difficult to achieve the regulation of the whole PANoptosis process by focusing on a single molecule. Here we discuss the regulation of PANoptosome which is central to PANoptosis.

Regulation of PANoptosome has two aspects: sensor expression level and content assembly. IRF1 is currently a key player in regulating PANoptosis at the gene level. During IAV infection, IRF1 upregulates ZBP1 expression upstream and promotes ZBP1‐PANoptosome formation, boosting cell death.^101^ Lack of IRF1 results in a marked blockage of the activation of ZBP1 and its downstream molecules caspase‐1/3/8 and MLKL.^102^ In IAV‐infected cells, the expression of IRF1 is primarily reliant on type I IFN. When cells lack IFN receptors such as INFAR1/2, ZBP1 expression is not upregulated and its mediated PANoptosis does not occur.^103^ This is in line with recent findings that diABZI, an agonist of the stimulator of interferon genes (STING), can induce neutrophil lung inflammation and PANoptosis.^104^

Transforming growth factor β activated kinase 1 (TAK1) was identified alongside the concept of PANoptosis as a regulator of the assembly of PANoptosome. Prof. Malireddi's team found that TAK1 could act as a regulatory master switch for the RIPK1 PANoptosome, as deletion or functional inactivation of *Tak1* could trigger the assembly of the RIPK1 PANoptosome. TAK1 inhibits the phosphorylation of RIPK1, limits its activation and prevents the spontaneous activation of PANoptosis. In 2020, the research team made an intriguing discovery that TAK1 deficiency could induce the activation of RIPK1‐independent PANoptosis, mediated by RIPK3‐MLKL. This finding was successfully validated through in vivo experiments, where the inactivation of Tak1 led to myeloproliferation and a severe sepsis‐like syndrome driven by the RIPK3‐caspase‐8 signaling axis. Notably, this entire process occurred without triggering a high expression of RIPK1.^18^, ^105^, ^106^ Adenosine deaminase acting on RNA1 (ADAR1), the only human protein besides ZBP1 that has a Zα domain, can also regulate PANoptosis.^107^ In the cytoplasm, it was found that ADAR1 could interact with ZBP1 through its Zα domain. By competing with RIPK3 for ZBP1, ADAR1 restricts ZBP1‐mediated PANoptosis.^108^ Caspase‐6, a component of the ZBP1‐PANoptosome mentioned before, also regulates its assembly. Under normal conditions, the association between ZBP1 and RIPK1 is stronger than its association with RIPK3. However, caspase‐6 can strengthen its association with ZBP1 by interacting with RIPK3.^86^ The mechanism of how caspase‐6 plays a role in the competitive relationship between RIPK1 and RIPK3 for ZBP1 remains to be explored, and one possible explanation is that caspase‐6 provides a convenient platform for the binding of RIPK3 and ZBP1.^77^

There are also many signaling axes that have a role in regulating PANoptosis. One of the regulatory points of the AIM2 PANoptosome is the cytoplasmic release of mtDNA, which has previously been shown to be an important trigger of AIM2 activation in pyroptosis.^109^ The latest study found that activation of the PANoptosome in a model of myocardial injury caused by doxorubicin was mainly mediated by the dsDNA receptor AIM2. Knockdown of the mitochondrial membrane protein gene *Fundc1*, which inhibits mtDNA cytoplasmic release, would activate the onset of PANoptosis.^110^ This suggests an essential regulatory role for mtDNA cytoplasmic release in the AIM2 PANoptosome. In addition to this, it has been found that during SARS‐CoV‐2 infection, TNF‐α and ITN‐γ produced in the organism lead to widespread PANoptosis, causing a lethal cytokine storm. Mechanistically, the combination of TNF‐α and ITN‐γ activates the JAK/STAT1/IRF1 axis, which induces nitric oxide production and triggers caspase‐8/FADD‐mediated PANoptosis. Moreover, the researchers were able to protect the organism from uncontrolled cell death during viral infection with a combined inhibitor of TNF‐α and ITN‐γ.^111^

## 5 PANOPTOSIS IN CANCER

The assembly of PANoptosome and the activation of PANoptosis is a self‐protective response by the body to effectively defend itself against foreign viral, bacterial, and fungal infections.^112^, ^113^ The concept of PANoptosis highlights the importance of molecular precision in disease therapeutics, as the boundaries between PCD pathways become increasingly blurred, and the therapeutic effect of blocking or interfering with a single mode of death may be unsatisfactory. Current approaches to blocking inflammatory cell death include knocking down targeted sensor molecules (e.g., NLRP3), inhibiting enzymatic targets (e.g., RIPK1), and neutralizing downstream cytokine signaling (e.g., IL‐1).^83^, ^114^, ^115^ However, these inhibitors are designed to specifically target one pathway in pyroptosis, apoptosis, and necroptosis, and clinical outcomes are currently not optimal. The emergence of PANoptosis has inspired the possibility of broadly blocking the three modes of death to which PANoptosis belongs by targeting upstream signaling pathways, receptors, or scaffold molecules, which undoubtedly opens up new therapeutic options for human‐related diseases.^20^

**FIGURE 4:**
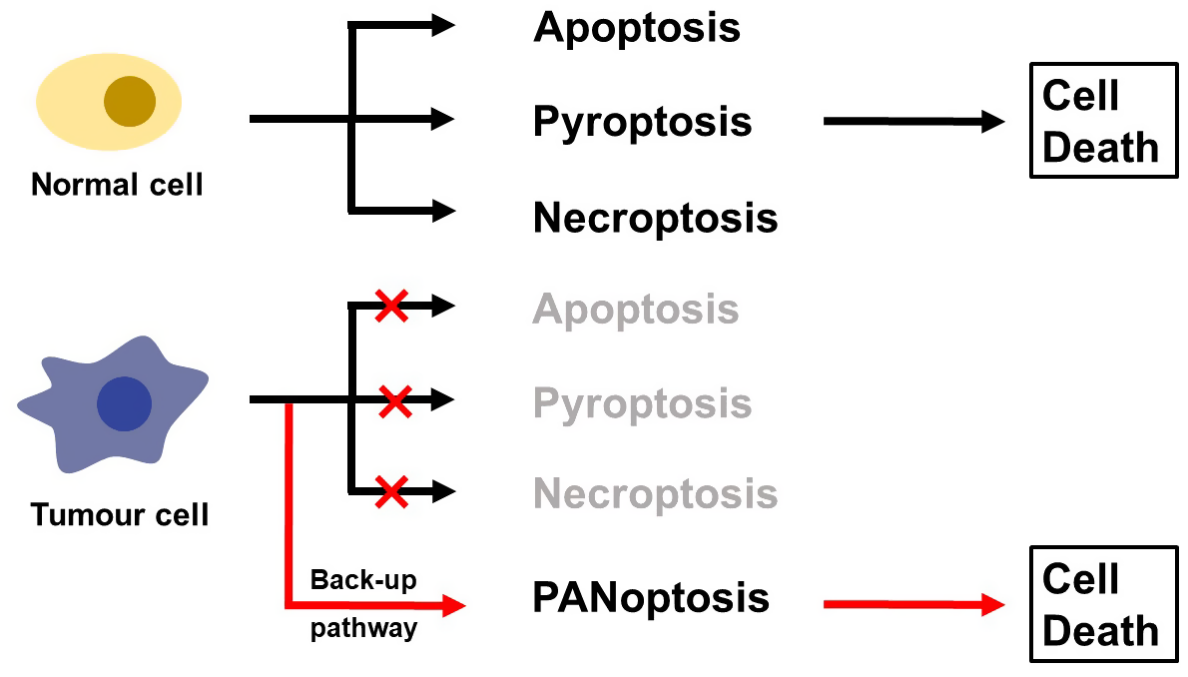
Relation between PANoptosis and cancer. Tumor cells often evade normal cell death pathways to proliferate uncontrollably. PANoptosis, in such cases, provides an alternative means of cell death, enabling tumor cells to eventually undergo cell death.

In contrast to the intensive research on PANoptosis in the field of infections, the development of PANoptosis in cancer is still in its infancy. Nevertheless, there is growing evidence that many similarities exist between tumor immunity and infection immunity, and that the role of PANoptosis in cancer cannot be ignored.^108^, ^116^ In contrast to the largely negative role of PANoptosis in inflammation, such as the induction of macrophage death and the release of large amounts of inflammatory factors, its role in the cancer environment is now mainstream considered to be beneficial.^117^, ^118^ Resistance to cell death is an important hallmark of cancer, and researchers believe that PANoptosis provides an alternative mechanism that can act as a “backup program” for cell death when one or more programmed cell death pathways are blocked in cancer cells^119^ (Figure 4). Table 1 summarizes the main finding of the studies of PANoptosis in cancer. Although the role of each of the three PCD pathways, apoptosis, pyroptosis, and necroptosis, in cancer has been discussed in detail, the specific molecular targets of PCD for anticancer therapy are challenging to identify, given the many overlaps and synergistic effects of the three death pathways. PANoptosis, on the other hand, provides a perspective on the overall biological effects of PCD to investigate and identify molecular functions and relevant therapeutic targets.^120^ TNF‐α and IFN‐γ have been found to induce PANoptosis in cells from 13 different human cancer cell lines, including colon cancer, lung cancer, and melanoma. Although the sensitivity of individual cancer cell lines to TNF‐α and IFN‐γ varied, the combination treatment was shown to have the ability to broadly induce cancer cell death.^118^, ^121^, ^122^

**TABLE 1:** The main finding of the studies of PANoptosis in cancer.

Cancer types	Conditions	Mechanism	Reference
Colorectal cancer	IRF1 stimulation	IRF1 induces PANoptosis in colon cells to protect them from tumor development.	102, 123
Colorectal cancer	*Adar1* knockout	*Adar1* knockout boosts ZBP1‐mediated PANoptosis.	87, 108, 125
Colorectal cancer	*Nfs1* knockout	*Nfs1* knockout enhances intracellular oxidative stress‐induced PANoptosis.	129, 130
Breast cancer	Prognostic model	PANoptosis prognosis‐related genes were prominently expressed in arterial endothelial cells.	^135^
Gastric cancer	PANscore model	Different scores provide distinct therapeutic options for the treatment.	^136^
Melanoma	High expression of *Zbp1*, *caspase‐8* and g*sdmd*	High expression of these genes induces ZBP1‐mediated PANoptosis in melanoma cells.	^138^
Colon adenocarcinoma	Transcriptome data	Nine lncRNAs were associated with COAD metastasis and PANoptosis.	^139^
Low‐grade gliomas	Gene clusters	Two gene clusters help predict the prognosis of gliomas.	^141^

### 5.1 Colorectal cancer

Karki and colleagues first explored the role of IRF1 in regulating tumorigenesis and cell PANoptosis. IRF1 was found to significantly reduce colorectal cancer incidence in mice, and its induction of PANoptosis was effective in preventing AOM/DSS‐induced colorectal cancer, while *Irf1*‐deficient mice exhibited an abnormal susceptibility to colitis‐associated colorectal tumors. The researchers compared the differences in pro‐inflammatory cytokine production and colonic cell death between *Irf1*‐deficient and wild‐type mice and found that they were similar in terms of pro‐inflammatory cytokine production, but that colonic cell death was significantly reduced in *Irf1*‐deficient mice. The researchers then further demonstrated that the reduction in colonic cell death was associated with impaired activation of the apoptosis protein caspase‐3/7 and reduced pyroptosis and necroptosis, possibly through the regulation of the secretion of TNF‐α and IFN‐γ by IRF1. This suggests that IRF1, as an upstream regulator of PANoptosis, induces PANoptosis in colon cells to protect them from tumor development, and that IRF1 may therefore be a potent target for the regulation of multiple programmed cell death.^102^, ^123^

ZBP1 serves as the most classical sensor of PANoptosome that induces cell death while adenosine deaminase acting on RNA 1 (ADAR1), which works as an RNA editor, maintains the balance between cell death and survival.^124^ One study described the interaction of ADAR1 with ZBP1, identifying its role in cell death regulation and tumor. The combination of interferon (IFN) and nuclear export inhibitor (NEI) activates ZBP1‐dependent PANoptosis, which ADAR1 can inhibit by interacting with the Zα2 structural domain of ZBP1 to limit the interaction between ZBP1 and RIPK3. *Adar1* knockout mice are resistant to the development of colorectal cancer, which can be reversed by deletion of the Zα2 domain of ZBP1. This finding suggests that ADAR1 inhibits ZBP1‐mediated PANoptosis and promotes tumor, providing information for therapeutic strategies for colorectal cancer or other diseases.^87^, ^108^, ^125^

Metabolic reprogramming is an important signature of malignant cells.^126^ Tumors frequently establish enduring reliance on the restructuring of metabolic functions, offering opportunities for tumor diagnosis, surveillance, and therapeutic interventions.^127^ Considering the indispensable role of metabolic enzymes in metabolic reprogramming, their aberrant expression is closely related to tumorigenesis, tumor progression, and chemotherapy sensitivity. Iron–sulfur (Fe‐S) clusters are important cofactors of Fe‐S proteins and are involved in a variety of cellular processes, including iron homeostasis, energy metabolism, and lipid biosynthesis.^128^ Fe‐S clusters are significantly increased during rapid growth of cancer cells. Using a CRISPR‐Cas9 screen of metabolic enzyme genes in vivo, it was found that deletion of cysteine desulfurase (*Nfs1*), the rate‐limiting enzyme in Fe‐S cluster biogenesis, enhances antitumor therapy by increasing intracellular oxidative stress‐induced PANoptosis in synergy with oxaliplatin, a first‐line treatment for colon cancer. NFS1 prevents PANoptosis in an S293 phosphorylation‐dependent manner under oxaliplatin treatment. The study also demonstrated that high expression of NFS1 in CRC patients is associated with poor prognosis.^129^, ^130^

### 5.2 Breast cancer

It has been demonstrated that TNF‐α exerts a downregulatory effect on the expression of the transcription factor AP‐2α in breast cancer cells, consequently inducing apoptosis.^131^ Additionally, the disruption of mitochondrial membrane integrity leads to the release of cytochrome c, which activates caspase‐3/6/9, ultimately triggering apoptosis in MCF‐7 breast cancer cell lines.^132^ Furthermore, in breast cancer, the NLRP3 inflammasome promotes the activation of GSDMD‐mediated pyroptosis by facilitating the release of IL‐1β and IL‐18.^133^ It is noteworthy that both bufalin and quercetin (Que) induce necroptosis in breast cancer cells by targeting the activation of RIPK1/3. These findings collectively suggest that all three death pathways in PANoptosis play significant roles in breast cancer pathogenesis.^134^

A recent study utilizing bioinformatics technology identified eight genes significantly associated with PANoptosis in breast cancer, including CXCL16. The researchers employed COX regression and LASSO regression to construct prognostic models, leading to risk stratification of patients. The results demonstrated that the low‐risk group exhibited a more favorable prognosis, characterized by higher levels of immune infiltration and immune checkpoint‐related gene expression. Moreover, the study confirmed that radixin (RDX), the gene with the highest hazard ratio (HR) among the PANoptosis prognosis‐related genes, was prominently expressed in arterial endothelial cells (ECs). This investigation enriches our comprehension of the involvement of PANoptosis in breast cancer and offers promising new directions for breast cancer immunotherapy.^135^

### 5.3 Other cancer

PANoptosis has been linked to many types of cancer. Researchers established three different patterns of PANoptosis by analyzing 1316 gastric cancer patients, each with unique clinical, molecular, and immunological features that can be quantified by a scoring system called PANscore. The different scores provide distinct therapeutic options for the treatment of gastric cancer.^136^

Another study found that high expression of PANoptosis genes is beneficial for melanoma, a potentially fatal form of skin cancer with a poor incidence of response to single‐agent chemotherapy.^137^ High expression of key molecules in PANoptosis such as *Zbp1*, *caspase‐8*, and *Gsdmd* was significantly and positively correlated with the survival probability and prognosis of melanoma patients. To validate the therapeutic effect of PANoptosis, the researchers successfully induced PANoptosis in melanoma cells by activating ZBP1.^138^

Huang et al. obtained transcriptome data from the TCGA and GEO databases for colon adenocarcinoma (COAD) and metastatic colon cancer to analyze differentially expressed genes. Single gene correlation analysis was also performed on 14 extensive apoptosis‐related genes to identify extensive apoptosis‐related lncRNAs, which were then analyzed together with differential genes from TCGA and GEO data to obtain nine lncRNAs, which were lncRNAs associated with COAD metastasis and PANoptosis. One lncRNA (SNHG7) was subsequently found to be significantly associated with tumor stage, lymph node metastasis, and prognosis of colon adenocarcinoma using bioinformatic analysis techniques.^139^ In addition, both drug‐sensitivity analysis and in vitro cellular assays showed that the lncRNA SNHG7 was involved in chemoresistance in COAD.

The poor prognosis of low‐grade gliomas (LGG) places a premium on biomarkers that can predict clinical outcome.^140^ Chen et al. developed a machine‐learning artificial neural network (ANN) to differentiate PANoptosis‐associated tumor subgroups by analyzing PANoptosis‐associated gene clusters (Cluster A and Cluster B), which can help to predict the prognosis of gliomas to some extent. LGGs with higher expression of cluster A usually have a poorer prognosis, whereas the opposite is true for LGGs with higher expression of cluster B.^141^ Interestingly, some key proteins in PANoptosis, such as caspase‐3/7/8, GSDMD, and RIPK1/3, are more expressed in cluster A, which seems to be contrary to the prevailing view that PANoptosis plays a positive role in tumors. Another research team independently discovered that elevated expression of PANoptosis genes had unfavorable effects in cases of LGG and kidney renal cell carcinoma (KIRC), while it conferred benefits in melanoma.^138^ Thus, PANoptosis does not always have an anticancer effect in all tumor types, and the mechanisms behind it remain to be investigated.

## 6 CANCER THERAPY THROUGH PANOPTOSIS

Apoptosis, pyroptosis, and necroptosis are extensively investigated programmed cell death (PCD) pathways in cancer therapy, aiming to impede cancer progression by influencing tumor cell death.^142^ Although drugs targeting individual PCD pathways have shown anticancer effects, they often fail to completely kill cancer foci due to potential evasion by cancer cells. By contrast, PANoptosis, which involves the activation of three death pathways, presents a promising approach to effectively eliminate cancer cells. Although there are no drugs that explicitly treat cancer through PANoptosis, there has been much progress in research targeting this area.^143^

The previously mentioned malignant melanoma is a typical representative cancer being studied for PANoptosis‐based cancer therapy. Even though the combination of metbine (MET) and doxorubicin (DOX) works well to treat many tumor types, including malignant melanoma, differences in their physicochemical properties and doses administered largely limit the effectiveness of the combination in tumors.^144^ A team of researchers has designed a biocompatible and tumor‐targeting precise folic acid‐cholesterol‐sodium alginate nanoparticle (FCA‐NP) that delivers MET and DOX to melanoma cells much more efficiently. In terms of currently known mechanisms, MET triggers PANoptosis by increasing the expression of N‐GSDMD and MLKL while DOX upregulating the expression of caspase‐7 and GSDMD. Melanoma cells underwent PANoptosis following drug treatment.^145^ Although the upstream receptors and other involved molecular mechanisms by which FCA‐NP carrying MET and DOX trigger PANoptosis in melanoma tumors are not completely clear, the establishment of such a system suggests that how to effectively induce PANoptosis may be a novel target for cancer treatment.

Notably, TNF‐α and IFN‐γ, as signaling axes regulating PANoptosis, can induce cell death in various human cancer cell lines, including lung, colon, and leukemia. This ability is not found in other cytokines.^118^, ^146^ Mechanistically, TNF‐α and IFN‐γ induce cancer cell death by activating crucial molecules in PANoptosis, such as GSDMD, caspase‐3/8/7, and MLKL. Combining intratumoral delivery of TNF‐α and IFN‐γ has been shown to reduce the weight and volume of xenograft tumors in mice, offering potential for future clinical applications. Moreover, ongoing phase III clinical trials (NCT00001296) investigating the combination of TNF‐α and IFN‐γ with hyperthermia isolated limb perfusion (HILP) in patients with locally advanced melanoma hold promise for achieving positive outcomes. Therefore, the synergistic effect of TNF‐α and IFN‐γ on PANoptosis in tumor cells may be a significant mechanism to inhibit tumor growth, providing new targets for anticancer therapies.

Therapies targeting PANoptosis also have the potential for combination with other cancer treatments. Immune checkpoint inhibitors (ICI) therapies, which target immune checkpoints on cancer cells and immune cells, can enhance the antitumor efficacy of various PCD pathways, including pyroptosis, necroptosis, and/or ferroptosis, even in ICI‐resistant cancers.^147^, ^148^ However, only a few studies have explored the combination of PANoptosis with ICIs, which could represent a novel opportunity for patients with refractory tumors. Chimeric antigen receptor T cell (CAR‐T) therapy is a cutting‐edge treatment option for antitumor therapy. However, cytokine release syndrome (CRS) induced by pyroptosis can diminish its efficacy.^149^ Similarly, CRS can be triggered by PANoptosis, and targeting specific pathways in PANoptosis to attenuate or block CRS would be a significant advancement for CAR‐T therapy.^117^

In summary, compounds targeted at PANoptosis for cancer treatment can be categorized into several aspects. Firstly, focusing on the upstream signaling pathways, such as TNF‐α and IFN‐γ. Upstream signaling pathways have a pivotal function in the regulation network of PANoptosis, and the advancement of antitumor drugs targeting these pathways holds significant promise. Secondly, centering on the receptors within PANoptosome. Receptors play a critical initiating role in PANoptosis, and drugs that can selectively enhance the expression of cell surface PANoptosis‐related receptors, such as ZBP1, are likely to exhibit favorable antitumor effects. Thirdly, targeting the assembly process of PANoptosome. The process is a complex endeavor, involving the assembly of various proteins through homotypic or heterotypic interactions between structural domains. Regulating the assembly of these proteins can also serve to control PANoptosis. Although there is currently limited research on drugs targeting this aspect, it is a direction worthy of exploration. Lastly, integrating PANoptosis with other advanced therapies presents a novel option for cancer treatment, although the underlying mechanisms require further investigation.

Despite the promising outlook of cancer therapies targeting PANoptosis, it is essential to note that treating cancer through PCD pathways and inflammatory signaling is a complex process closely tied to the specifics of the cancer type.^100^, ^146^ Therefore, selecting appropriate molecular targets for each specific cancer is critical, as different PCD pathways may perform different roles in various cancers. Moreover, since the target proteins and pathways for PANoptosis‐based therapies also play roles in apoptosis, pyroptosis, and necroptosis, it is essential to investigate whether these therapies, when regulating PANoptosis, might impact the pathophysiology of apoptosis, pyroptosis, and necroptosis. But in any case, PANoptosis represents a new research direction in cancer therapy.

## 7 CONCLUSION AND FUTURE PERSPECTIVES

PANoptosis represents one of the most complex forms of programmed death currently available and is closely linked to various human diseases, and its important role in cancer is gaining traction. Tumor cells exhibit an unending propensity to proliferate, resisting death to surmount growth restrictions and elude immune cell attacks. With the discovery of more ways of programmed death and the elucidation of related molecular mechanisms, our comprehensive of cell death in tumors constantly evolves. As multiple forms of cell death occur simultaneously in tumors, future studies on the interactions between multiple cell death processed in tumor development will aid in unraveling the pathogenesis of cancer and furnish valuable insights and tools for the development of relevant antitumor medications. The synergistic application of multiple programmed cell death modes in the context of cancer for their collective antitumor effects is also an essential avenue for future therapeutic exploration.^150^

Although impressive progress has been made in the study of PANoptosis, many questions remain to be addressed. For example, there is indeed cross talk between apoptosis, pyroptosis, and necroptosis, but is there a more comprehensive regulatory network? Viral, bacterial, and tumor cells can all induce PANoptosis via ZBP1 receptors, but what are the links and differences between them? Pathogens and tumor cells seek to escape PANoptosis to avoid their own elimination, but what are the molecular mechanisms by which these pathogens and tumor cells achieve the PANoptosis escape process? In addition to the four known PANoptosomes in the paper, are there other unidentified proteins or molecules that mediate the assembly of PANoptosome? Although the mainstream view is that PANoptosis is beneficial in cancer, some studies have found that it may be detrimental to certain types of tumors. Although there are still many questions about PANoptosis, the development of this concept and the related research progress have certainly opened a whole new field for researchers, and the early elucidation of the role and regulatory mechanism of PANoptosis in human diseases may lead to a new therapeutic strategy and a breakthrough in the treatment of patients concerned.

## AUTHOR CONTRIBUTIONS

**Hantao Cai:** Conceptualization (equal); investigation (lead); visualization (lead); writing – original draft (lead); writing – review and editing (lead). **Tingting Wang:** Conceptualization (lead); funding acquisition (lead); resources (lead); supervision (lead); validation (lead). **Mingming Lv:** Conceptualization (equal); investigation (equal); supervision (supporting).

## CONFLICT OF INTEREST STATEMENT

The authors declare that they have no competing interests.

## Data Availability

Data sharing not applicable to this article as no datasets were generated or analysed during the current study.
